# Enhanced antiproliferative activity of antibody-functionalized polymeric nanoparticles for targeted delivery of anti-miR-21 to HER2 positive gastric cancer

**DOI:** 10.18632/oncotarget.18066

**Published:** 2017-05-22

**Authors:** Feng-Lei Wu, Jian Zhang, Wei Li, Bao-Xiang Bian, Yi-Dong Hong, Zi-Yan Song, Hui-Yu Wang, Fang-Bo Cui, Ru-Tian Li, Qin Liu, Xiao-Dong Jiang, Xiao-Min Li, Jun-Nian Zheng

**Affiliations:** ^1^ Department of Onology, Affiliated Lianyungang Hospital of Xuzhou Medical University, Lianyungang, Jiangsu 222000, China; ^2^ Department of General Surgery, Huai'an First People's Hospital, Nanjing Medical University, Huai’an, Jiangsu 223000, China; ^3^ Center of Research Laboratory, Affiliated Lianyungang Hospital of Xuzhou Medical University, Lianyungang, Jiangsu 222000, China; ^4^ Department of Medicine, Jiangsu University, Zhenjiang, Jiangsu 212001, China; ^5^ Department of Oncology, Wuxi People's Hospital Affiliated to Nanjing Medical University, Wuxi, Jiangsu 214000, China; ^6^ Department of Oncology, The People's Hospital of Ma’anshan, Ma’anshan, Anhui 243000, China; ^7^ The Comprehensive Cancer Centre of Drum Tower Hospital, Medical School of Nanjing University, Clinical Cancer Institute of Nanjing University, Nanjing, Jiangsu 210008, China; ^8^ Department of Emergency, Affiliated Lianyungang Hospital of Xuzhou Medical University, Lianyungang, Jiangsu 222000, China; ^9^ Cancer Institute, Xuzhou Medical University, Xuzhou, Jiangsu 221002, China

**Keywords:** microRNA, gastric cancer, HER2, trastuzumab, nanoparticles

## Abstract

MiR-21 is an oncogenic miR frequently elevated in gastric cancer. Overexpression of miR-21 decreases the sensitivity of gastric cancer cells to trastuzumab, which is a humanized monoclonal antibody targeting human epidermal growth factor receptor 2. However, optimization of miRNA or its anti-miRNA oligonucleotides (AMOs) for delivery is a challenge. Receptor-mediated endocytosis plays a crucial role in the delivery of biotherapeutics including AMOs. This study is a continuation of our earlier findings involving poly(ε-caprolactone) (PCL)-poly (ethylene glycol) (PEG) nanoparticles (PEG-PCL NPs), which were coated with trastuzumab to target gastric cancer cells with HER2 receptor over-expression using anti-miRNA-21 antisense oligonucleotides (AMO-21). The antibody conjugates (HER-PEG-PCL NPs) act against target cells via antibody-dependent mechanisms and also based on encapsutalated AMO-21. X-ray photoelectron spectroscopy validated the presence of trastuzumab on NP surface. Sodium dodecyl sulfate-polyacrylamide gel electrophoresis (SDS-PAGE) revealed a stable antibody expression. The cell line specificity, cellular uptake, AMO-21 delivery, and cytotoxicity of the HER-PEG-PCL NPs were investigated. We found that the antibody conjugates significantly enhanced the cellular uptake of NPs. The HER-PEG-PCL NPs effectively suppressed the target miRNA expression in gastric cancer cells, which further up-regulated phosphatase and tensin homolog (PTEN). As a result, the sensitivity of HER2-expressing gastric cancer cells to trastuzumab was enhanced. The approach enhances the targeting by trastuzumab as well as antibody-dependent cellular cytotoxicity of immune effector cells. The antitumor effects of AMO-21-HER-PEG-PCL NPs were compared with trastuzumab in xenograft gastric cancer mice. The results provide insight into the biological and clinical potential of targeted AMO-21 delivery using modified trastuzumab for gastric cancer treatment.

## INTRODUCTION

Gastric cancer (GC) ranks fourth among all cancers in worldwide incidence. It ranks second in cancer-related deaths reported in China [1]. Most patients are diagnosed with surgically advanced or metastatic disease [2]. Despite the advantages of combination chemotherapy, the prognosis is poor. The 5-year survival of patients at advanced stages is around 5% to 20%, and the average overall survival time is less than 1 year [2, 3]. Approximately 7% to 34% of gastric cancers are characterized by poor prognosis associated with amplification of human epidermal growth factor receptor 2 gene (HER2) [4–6]. Treatment with a combination of trastuzumab, a humanized monoclonal antibody against HER2 and chemotherapy is indicated for HER2-positive advanced GC. Despite a significant survival advantage with the combination treatment, 12% of all HER2-positive GC cases show cancer progression [7].

MicroRNAs (miRs) are well known for suppression of translation via RNA-induced silencing complex (RISC). Changes in miRNA expression profile is correlated with tumor pathogenesis, cancer progression, and drug resistance [8]. Therefore, anti-miRNA oligonucleotides (AMOs) facilitate tumor therapy by abrogating the expression of specific miRs [9–12]. Down-regulation of miR-21, a gastric oncomiR, enhances the sensitivity of HER2-positive GC *in vitro* via apoptosis, in response to trastuzumab therapy [13]. Thus, targeted AMO-21 delivery improves the efficacy of trastuzumab.

However, AMO is sensitive to nucleases. It is hydrophilic with poor membrane permeability. The stability and efficiency of AMO therapy is increased by treatment with 2’-O-methyl or 2’-O-methoxyethyl agents [14]. The modifications may reduce the specificity and affinity for the target miRNAs [15], interfering with the efficient delivery of AMOs into cells. Therefore, enhancing the delivery of AMO using effective carrier vectors or Lipofectamine 2000 has been shown to be effective in proof-of-concept experimental studies. Nevertheless, viral vectors are potentially oncogenic, triggering immune response, inducing viral mutations and possess limited loading capacity [16]. Lipofectamine 2000 is potentially cytotoxic, non-specific for tissues, and is sensitive to serum proteins [16]. Safe and effective nonviral delivery systems are needed for clinically viable cancer treatment. Polymeric nanoparticle-based delivery is a promising strategy with high transfection efficiency. Polymeric nanoparticles are easily modified and functionally enhanced, and delivered safely without any toxicity [17]. Therefore, the design of a targeted nanoparticle system could overcomes the disadvantages of AMO effectively.

**Figure 1:**
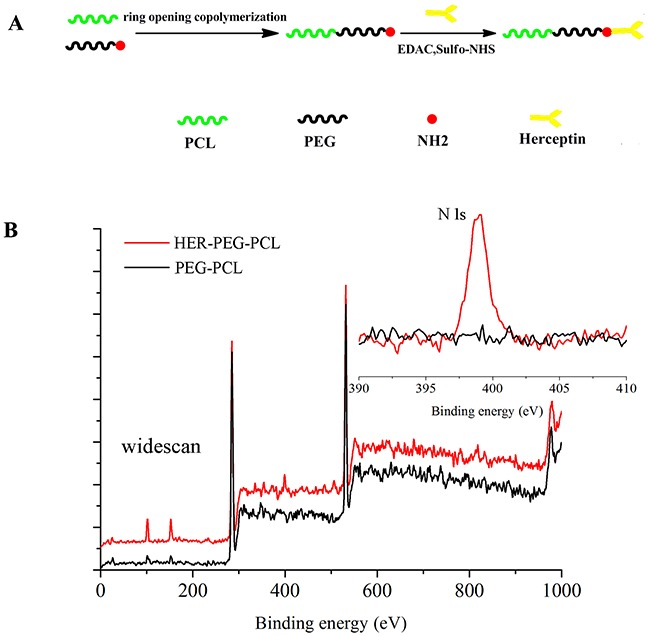
**(A)** Schematic illustrating the fabrication of trastuzumab-conjugated PEG-PCL NPs: the NPs comprise a PCL core, a hydrophilic and stealth PEG shell on the surface of the core and a herceptin ligand coating. **(B)** Representative XPS spectrum and N 1s peak (the inset) of the HER-PEG-PCL nanoparticles before (lower curve) and after trastuzumab conjugation (upper curve).(NPs: nanoparticles; AMO-21: anti-miR-21 oligonucleotide; XPS: X-ray photoelectron spectroscopy).

Nanodelivery systems have been shown to overcome the challenges posed by AMOs in therapeutic drug delivery in cancer using miRNA interference [18, 19]. The challenges of effective gene silencing relate to active recognition of target cells, without affecting normal cells [20]. Complexation of NPs with target-specific ligands or antibodies enhances therapeutic efficacy by facilitating targeted delivery to cancer cells [21, 22]. Based on our previous studies, we conjugated trastuzumab with poly(ethyleneglycol) (PEG) and poly(ε-caprolactone) (PCL) copolymers [23], using PEG as the linker molecule (HER-PEG-PCL). Receptor mediated targeting of gastric cancer (GC) cells was accomplished by overexpressing HER2, using precision engineering of nanoparticles of biodegradable copolymers to quantitatively control AMO-21 delivery (Figure 1A). The approach enhances the targeting by trastuzumab as well as antibody-dependent cellular cytotoxicity (ADCC) of immune effector cells. We have controlled the surface ligand density of molecules using copolymers and trastuzumab in therapeutically effective ratios. The AMO-21 was delivered by HER-PEG-PCL (AMO-21-TNPs) to GC cells overexpressing NUGC4 and not SGC7901. The AMO-21-loaded PEG-PCL nanoparticles (AMO-21-NPs) that failed to target HER2, and Lipofectamine 2000 were used as controls. We hypothesized that trastuzumab combined with AMO-21 was a promising therapeutic strategy for cancer. Enhanced trastuzumab cytotoxicity in cancer cells is mediated by the antagonism of target miR-21 against increased expression of PTEN. Thus, multifunctional nanoparticles may represent a generalized approach for the treatment of GC.

## RESULTS

### Preparation of HER-PEG-PCL copolymer

A single-step carbodiimide technique was employed to synthesize HER-PEG-PCL copolymers. The method was based on the PEG-PCL synthesized as described in Materials and Methods. Activation of carboxyl groups on the antibody molecules was followed by reaction with the primary amino groups on the PEG-PCL chains. The resulting amide bonds link the antibodies on the NPs surface. The antibody conjugation of NPs was confirmed by analyzing their surface chemistry using XPS to identify the changes in nitrogen signal according to the specific binding energy. Trastuzumab containing 1726 nitrogen atoms emits stronger signals than the amino groups in the PEG-PCL molecules. Distinct signal peaks emitted from nitrogen (N 1s) suggests antibody conjugation in the polymeric matrix cores although the non-conjugated NPs also present lower signals associated with nitrogen in the amino groups on the surface. Therefore, the results validate successful conjugation of antibody molecules with the polymer matrix (Figure 1B).

### Ligand surface density

We investigated the relationship between antibody conjugates on the NPs surface with the PEG-PCL-to-trastuzumab ratio. A series of weight ratios (w/w) of PEG-PCL/trastuzumab 20%, 40%, 60% and 80% were used to prepare HER-PEG-PCL copolymers. The final levels of trastuzumabconjugated to the NPs surface were: 0.077, 0.236, 0.229, and 0.242 mg per mg of NPs after subtracting the background levels using 0% NPs as control (Table 1). Therefore, 40% weight ratio (w/w) of PEG-PCL/trastuzumab was selected to synthesize HER-PEG-PCL copolymers for all subsequent experiments.

**Table 1:** Trastuzumab content of the HER-PEG-PCL nanoparticles of various PEG-PCL amounts used in the nanoprecipitation process

Ratio of PEG-PCL to Trastuzumab (% w/w)	Trastuzumab content (% w/w)
20	7.7 ± 0.58
40	23.6 ± 1.8
60	22.9 ± 1.56
80	24.2 ± 2.09

^a^ The SD value was for the mean trastuzumab content (% w/w) obtained from the three measurements.

### SDS-PAGE

Trastuzumab is inactivated by irreversible den-aturation or aggregation during NP synthesis. Trastuzumab bound to the NP surface was extracted and subjected to SDS-PAGE. Trastuzumab extracted from HER-PEG-PCL NPs under reducing conditions was compared with native protein (Lane 3) and the protein conjugated with PEG-PCL NPs (Lanes 4) (Supplementary Figure 1). The protein remained constant after coating the NP surface, which validates the strategy using HER-PEG-PCL NPs for HER2-overexpressed cancer targeting.

### Preparation and characterization of anti-miR-21-loaded nanoparticles

**Figure 2:**
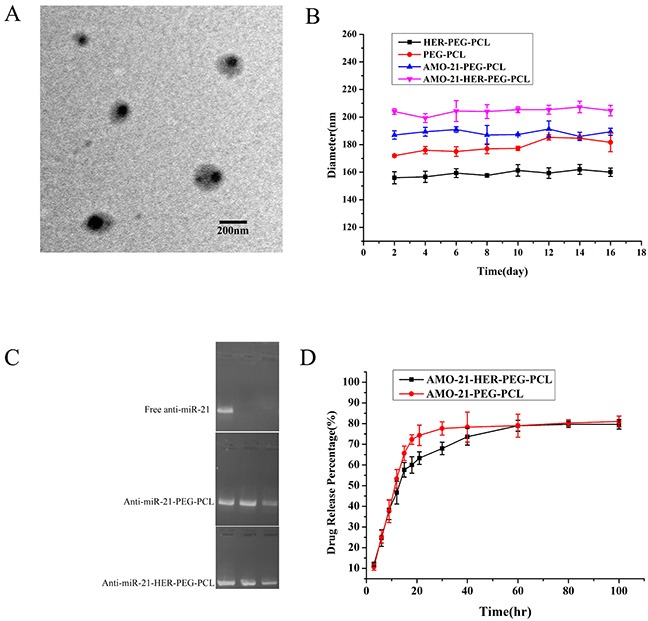
Characterization of AMO-21-HER-PEG-PCL **(A)** Morphology of AMO-21-HER-PEG-PCL by TEM. Scale bar represents 200 nm. **(B)** Stability of the NPs. The diameter of the NPs was determined by DLS and each value represents the mean ± SD. **(C)** Serum stability of AMO-21-HER-PEG-PCL and AMO-21 alone, AMO-21-HER-PEG-PCL, or AMO-21 mixed with 50% FBS at 37°C for 0 h, 6 h, and 12h. Samples were analyzed using gel electrophoresis. **(D)** The *in vitro* release of AMO-21. The data are presented as mean ± SD.(AMO-21: anti-miR-21 oligonucleotide; TEM: transmission electron microscope; NPs: nanoparticles; DLS: Dynamic light scattering).

AMO-21-NPs and AMO-21-TNPs were synthesized using a double-emulsion solvent evaporation technique as described earlier. NPs devoid of drug served as controls (NPs control). The average NPs ranged from 176.3 to 213.3 nm in size, which facilitated the enrichment of NPs in the tumor tissue by increasing the permeability and retention (EPR) [24] The zeta potentials were negative, ranging from −7.27 mV to −11.23 mV, and the polydispersity ranged from 0.181 to 0.268 (Table 2). TEM (Figure 2A) images revealed that AMO-21-TNPs were spherical, measuring approximately 200 nm in average diameter. The DLCs of AMO-21 in AMO-21-NPs and AMO-21-TNPs were 0.38 ± 0.016% and 0.34 ± 0.042%, respectively. The EE of AMO-21 in AMO-21-NPs and AMO-21-TNPs were 53.12 ± 2.3% and 50± 4.2%. The trastuzumab content of TNP and AMO-21-TNP was 19.24± 4.49 % and 21.18 ± 2.23%, respectively. (summarized in Table 2). This AMO-21-TNPs formulation yielded a trastuzumab-to-AMO-21 mass ratio of 62.6:1.

**Table 2:** Mean particle size and drug load efficiency of four kinds of nanoparticles

Nanoparticles	Diameters (nm)^a^	Polydispersity^a^	Zeta potential (mV)^a^	AMO-21 DLC (%) AMO-21 EE (%)	Trastuzumab content (% w/w)
PEG-PCL	176.3 ± 6.6	0.195 ± 0.070	−8.90 ± 1.47	-	-
PEG–PCL-AMO21	189.0 ± 4.5	0.268 ± 0.008	−7.27 ± 1.91	0.38 ± 0.01653.12 ± 0.023%	-
HER2-PEG -PCL	202.3 ± 7.6	0.181 ± 0.079	−8.54 ± 1.59	-	19.24± 4.49 %
HER2-PEG-PCL-AMO21	213.3 ± 8.7	0.242 ± 0.038	−11.23 ± 0.95	0.34 ± 0.04250± 0.042%.	21.18 ± 2.23%

^a^ The SD value was for the mean particle size obtained from the three measurements.

DLC: drug loading content.

### Structural stability, AMO-21 protection and release from TNPs *in vitro*

Particle sizes of AMO-21-TNPs and AMO-21-NPs were stable for more than a fortnight (Figure 2B). Serum stability was used to determine the ability of TNPs and NPs to protect anti-miR. In this study, free AMO-21, AMO-21-PEG-PCL and AMO-21-HER-PEG-PCL were incubated with FBS at 37°C for different time periods. As shown in Figure 2B, PEG-PCL and HER-PEG-PCL NPs protected AMO-21 against nuclease activity for up to 12 h. The free AMO-21 was completely digested within 6 h, The results suggest that PEG-PCL and HER-PEG-PCL NPs were stable in the serum. AMO-21 was released in a burst from TNPs and NPs during the first 10 h, followed by a sustained release over the next 96 h (Figure 2D).

### Cellular uptake

**Figure 3:**
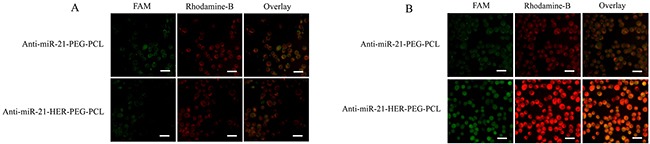
Fluorescence images of AMO-21-PEG-PCL and AMO-21-HER-PEG-PCL NPs in NUGC4 cells **(A)** and SGC7901 cells **(B)**. AMO-21 was labeled with FAM (green) and NPs were labeled with the fluorescent rhodamine-B (red). Scale bar: 100 μm (NPs: nanoparticles; AMO-21: anti-miR-21 oligonucleotide).

We investigated the cellular uptake of HER-PEG-PCL nanoparticles based on HER2 expression. The rhodamine-labeled FAM-AMO-21-TNPs and FAM-AMO-21-NPs were used to track the position of nanoparticles. As shown in Figure 3A, 3B, the green fluorescence emitted by FAM-AMO-21 and the red color from rhodamine B-labeled nanoparticles merged in the cytoplasm, suggesting entry of AMO-21 into cytosol together with nanoparticles. HER2-overexpressing NUGC4 cells were used as a positive control to compare the SGC7901 cells with negative HER2 expression. In SGC7901 cells, the IOD of Rhodamine B and FAM did not differ significantly between groups (PEG-PCL, HER-PEG-PCL) (P > 0.05). However, the IOD of Rhodamine B and FAM in the HER-PEG-PCL group were significantly higher than the PEG-PCL group in NUGC4 cells (P < 0.001) (Supplementary Table 2), the IOD of Rhodamine B and FAM in the PEG-PCL group indicated similar cellular uptake in both NUGC4 and SGC7901 GC cells (P > 0.05), (Supplementary Table 3), suggesting effective targeting of HER-PEG-PCL nanoparticles into NUGC4 cells.

### *In vitro* cytotoxicity and apoptosis

**Figure 4:**
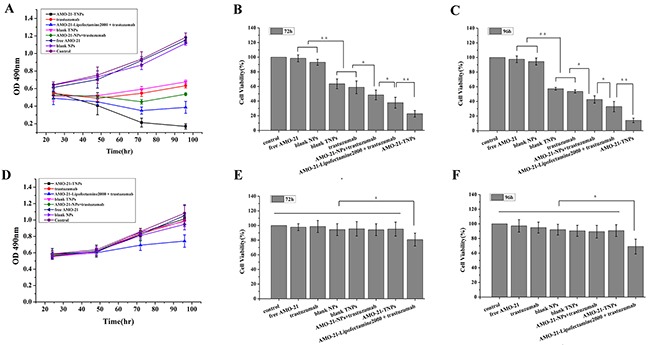
Effect of AMO-21(100 nmol/L), trastuzumab (43 μg/mL), blank NPs, blank TNPs, AMO-21-NPs, AMO-21-TNPs, AMO-21-Lipofectamine 2000 + trastuzumab, and AMO-21-NPs + trastuzumab on the growth of human NUGC4 **(A)** and SGC7901 cells **(D)**. Cell growth was determined by MTT at every 24 hours for 4 days. Saline was used as treatment control. Cell viability was detected in both gastric cancer cells at 72 h **(B, E)** and 96 h **(C, F)** of treatment (*P < 0.05; **P < 0.01) (AMO-21: anti-miR-21 oligonucleotide; NPs: PEG-PCL nanoparticles; TNPs: HER-PEG-PCL nanoparticles).

The *in vitro* antitumor activity of the formulations were analyzed in NUGC4 and SGC7901 cells after treatment with saline (saline control), blank NPs, free trastuzumab (43 μg/mL), blank TNPs (equal trastuzumab concentration), free-AMO-21 (100 nmol/L), AMO-21-NPs (equal AMO-21 concentration), AMO-21-TNPs (equal concentrations of AMO-21 and trastuzumab), AMO-21-Lipofectamine 2000 + trastuzumab and AMO-21-NPs + trastuzumab for 4 days using MTT assay Cells treated with blank NPs exhibited maximum viability. It was similar to that of controls even after four days of incubation, suggesting absence of cytotoxicity (Figure 4A, 4B and 4C). NUGC4 cells treated with TNPs showed similar levels of cytotoxicity to trastuzumab. These cells showed higher cytotoxicity when treated with AMO-21-TNPs compared with free trastuzumab, AMO-21+trastuzumab and AMO-21-NPs + trastuzumab. No significant changes in cell viability were observed between treated and untreated SGC7901 cells except for AMO-21-Lipofectamine 2000 + trastuzumab (Figure 4D, 4E and 4F).

### AMO-21-TNPs enhance apoptosis of NUGC4 cells

**Figure 5:**
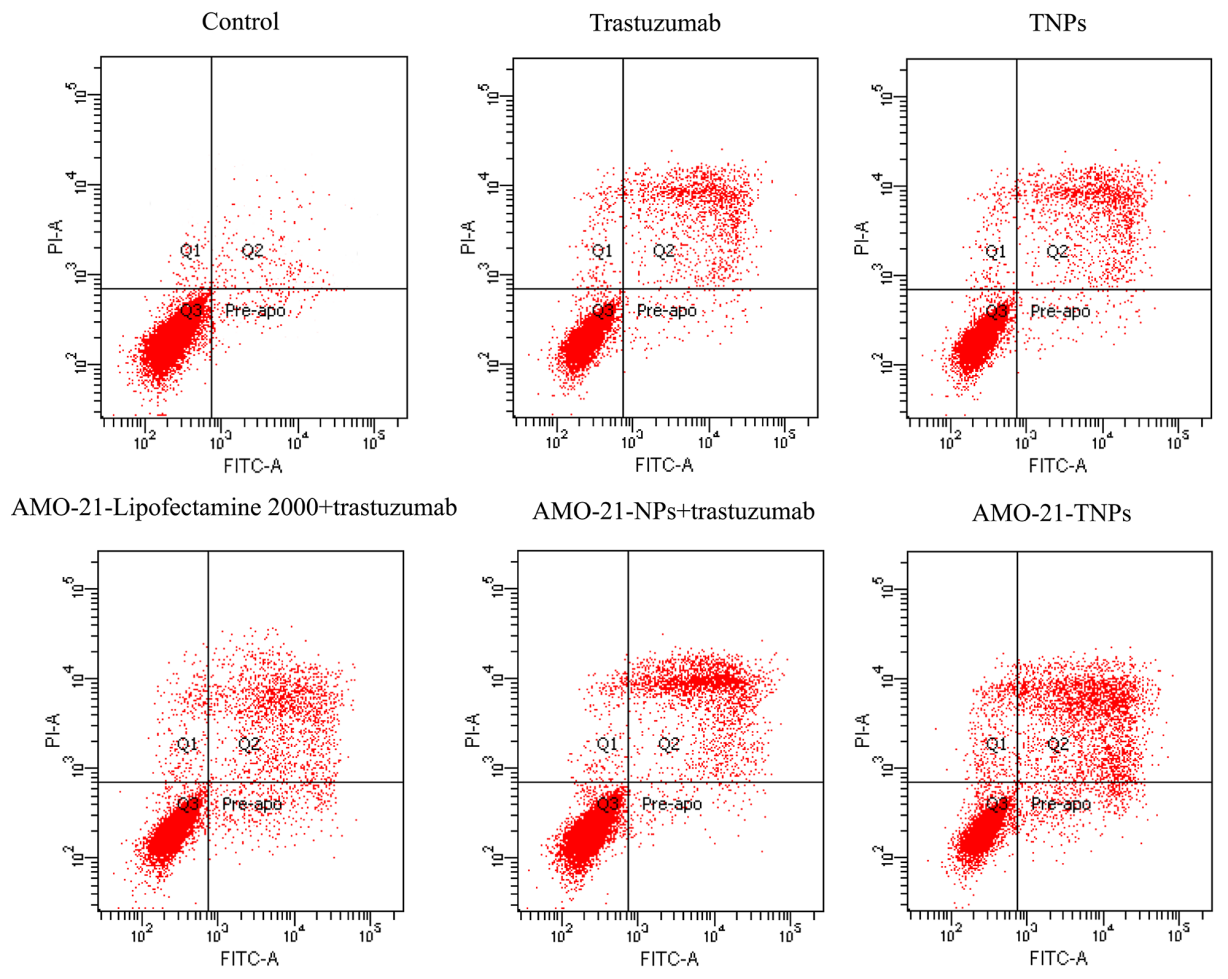
Apoptosis of NUGC4 after exposure to saline, trastuzumab, blank TNPs, AMO-21-Lipofectamine 2000 + trastuzumab, AMO-21-NPs + trastuzumab, and AMO-21-TNPs for 72 hours The cells were stained with Alexa Fluor 488 annexin-V and PI (Life Technologies, Carlsbad, CA, USA), followed by flow cytometry at 72 h after treatment. Cellular incorporation of trastuzumab and blank TNPs slightly increased the fraction of apoptosis compared with saline control (14.43 ± 1.22%, 26.43 ± 2.22%, 29.93 ± 3.03 %, 8.05± 0.79%, 10.19 ± 0.93%, respectively). The number of apoptotic cells treated with AMO-21-TNPs was elevated compared with control and other groups (63.68 ± 3.06%, *P* < 0.01). (AMO-21: anti-miR-21 oligonucleotide; NPs: PEG-PCL nanoparticles; TNPs: HER-PEG-PCL nanoparticles).

We measured the percentage of apoptotic cells 72 h after treatment with saline control, free trastuzumab, TNPs, AMO-21-Lipofectamine 2000 + trastuzumab, AMO-21-NPs + trastuzumab, and AMO-21-TNPs. As shown in Figure 5, a higher number of apoptotic cells was observed in the AMO-21-TNPs group (63.01 ± 2.4%) compared with cells treated with saline control (9.67 ± 8.4%), trastuzumab (29.93 ± 3.04%), TNPs (26.43 ± 2.22%), AMO-21-Lipofectamine 2000 + trastuzumab (51.59 ± 1.61%) or AMO-21-NPs + trastuzumab (39.01 ± 2.5%) (P < 0.01).

### Real-time NIRF imaging

NIR fluorescence signals were observed in both the tumors and the abdomen at 4 h post-intravenous administration (Supplementary Figure 4A). As time passed, the fluorescence density in the abdomen decreased, but the NIRF signal gradually increased in the tumor. At 144 h post-intravenous administration, a strong fluorescence signal could only be observed in the tumor region. NPs that were not located in tumor zones were likely to be eliminated from the liver, spleen, kidney, and other important tissues. The tumor, heart, brain, liver, spleen, kidneys, stomach, intestines, and the lungs were isolated to evaluate the tissue distribution of NPs at 144 h post-intravenous administration (Supplementary Figure 4B). The NIRF signal in the tumor was much higher than that in the other organs.

### *In vivo* antitumor activity of AMO-21-TNPs

Blank NPs and AMO-21-NPs did not inhibit tumor growth. The tumors treated with TNPs and trastuzumab were significantly suppressed compared with those treated with saline (P < 0.01), but the differences between TNPs and trastuzumab were not significant (P > 0.05). The AMO-21-TNPs began to show an antitumor effect greater than that of trastuzumab after the first 7 days. The antitumor advantages of AMO-21-TNPs became more prominent as time passed (P < 0.01). AMO-21-TNPs completely halted tumor growth, and its antitumor effect was much higher than that of TNPs and trastuzumab (P < 0.01). The tumors in the group treated with AMO-21-TNPs were the smallest among all groups (P < 0.01). Notably, the tumor suppressive effect of AMO-21-NPs was not significantly different from that of saline (P > 0.05) (Supplementary Figure 5). This is because miR-21 mostly plays a regulatory role in cancer cell proliferation.

### MiR-21 silencing and PTEN expression enhanced by AMO-21

**Figure 6:**
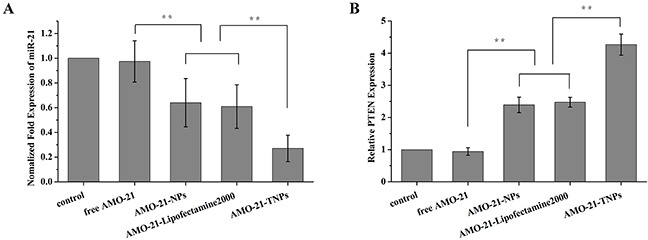
Expression of miR-21 and its target genes *PTEN* in NUGC4 cells after different treatments (from left to right, saline control, free AMO-21, AMO-21-NPs, AMO-21-Lipofectamine 2000, and AMO-21-TNPs) after 72 h The expression of miR-21 was normalized to U6 small nuclear RNA gene (U6 snRNA) control. The expression of *PTEN* was normalized to beta-actin. **(A)** Compared with the saline control, the expression of miR-21 in free AMO-21, AMO-21-NPs, AMO-21-Lipofectamine 2000 and AMO-21-TNPs was 97.5% (*P* > 0.05), 60.9%, 64.1% and 27.1%. (**P < 0.01). **(B)** Compared with saline control, free AMO-21, AMO-21-NPs, AMO-21-Lipofectamine 2000, AMO-21-TNPs up-regulated PTEN expression by 1.1-fold (*P* > 0.05), 2.4-fold, 2.5-fold and 4.2-fold (**P < 0.01) (AMO-21: anti-miR-21 oligonucleotide; NPs: PEG-PCL nanoparticles; TNPs: HER-PEG-PCL nanoparticles).

We investigated whether the encapsulated AMOs controlled the expression of target miRNAs and their downstream targets. NUGC4 cells were treated with AMO-21 containing TNPs and other controls for 4 h. The expression of miR-21 and its target gene were assayed 48 h after transfection using real time RT-PCR. As shown in Figure 6A, the miR-21 expression in the positive control, treated with Lipofectamine 2000, was 60.9% compared with that of the untreated cells. The miR-21 expression of AMO-21-NPs-treated groups was similar (64.1%). Notably, AMO-21-TNPs treated groups exhibited the lowest miR-21 expression (27.1%) of all the groups. PTEN is regulated by miR-21, and miR-21 inhibition alters the PTEN expression [13]. As shown in Figure 6B, we found a 4.2-fold PTEN up-regulation the 2.5-fold up-regulation of PTEN in the AMO-21-Lipofectamine 2000 and 2.3-fold PTEN up-regulation in the AMO-21-NPs groups, respectively. In contrast, no change in gene expression of free AMO-21 was found. In addition, the results indicated the superiority of TNP's over other transfection agents commercially available.

## DISCUSSION

We developed a novel trastuzumab-conjugated nanoscale drug delivery vehicle, with distinct therapeutic mechanisms based on the antibody *per se* (e.g., ADCC, CDC) and the carrier molecule AMO-21. We initially demonstrated therapeutic applications of trastuzumab conjugated with nanoparticles encapsulated with AMO-21 against GC cells. The nanoparticle-based antibody conjugates not only increased the efficiency of targeting and AMO-21 transfection efficiency, but also sensitized the cancer cells to trastuzumab resulting in effective tumor suppression.

MiRs control key genes involved in cancer and drug resistance [25]. MiR-21 is a potential oncogenic miR involved in gastric cancer and is associated with trastuzumab resistance [13]. The miR overexpression is silenced by anti-miRs in a targeted manner. AMO-21 was developed to specifically target miR-21. Traditional methods of oligonucleotide delivery are mediated via viruses and lipids such as Lipofectamine 2000. Modification of the oligonucleotide backbone also increases the stability of the miRs against enzymatic degradation and facilitates direct delivery to cells without the need for transfection. However, these methods are limited by their efficacy *in vivo* by serum nucleases, lysosomal degradation, and non-specific absorption.

A nanoparticle-based delivery system improves stability and delivery efficiency. NPs-based delivery may be via active or passive targeting [26]. Passive targeting is facilitated by EPR or via localized application and delivery. Active targeting is accomplished by conjugating different targeting moieties with the surface of NPs [27]. Antibodies represent ideal anticancer agents. They have been the focus of intense research activity since the production of customized monoclonal antibody in the mid-1970s. However, antibodies have been used successfully as immunotargeting agents [28]. Our strategy employed tumor-cell-specific NPs conjugated with antibodies such as trastuzumab with the NP surface. We previously reported effective delivery of docetaxel and miRNA using PEG-PCL-based nanoparticles. PEG-PCL-based nanosystems targeted cancer cells and enhanced the cytotoxicity of docetaxel by sequence-specific gene silencing [29]. Our current study developed nanoparticles of copolymers PEG-PCL conjugated with trastuzumab for targeted delivery of AMO-21. HER-PEG-PCL NPs were prepared using one-step carbodiimide coupling using EDAc and Sulfo-NHS in aqueous phase. Covalent coupling of amino groups at the surface of PEG-PCL with the carboxyl groups of trastuzumab was analyzed by XPS (Figure 1B). The formulation with the highest surface density of the ligand was further evaluated for the delivery of AMO-21 into GC cells. A herceptin-to-PEG-PCL mass ratio of 1:1 favored efficient conjugation (Table 1). The free amino groups on the NPs surface provide linkers for the ligand molecules in the presence of EDAc. The increase in the number of amino groups in the PEG-PCL molecules increased the efficiency of covalent coupling. At a 50% ratio of PEG-PCL-to-trastuzumab, the binding efficiency was the highest. SDS-PAGE revealed stability and activity of the antibody after nanoparticle synthesis (Supplementary Figure 1). The NPs generated by the double-emulsion solvent evaporation measured approximately 200 nm in size, with a negetive zeta potential conducive to cellular uptake via endocytosis [24] (Figure 2B and Table 2). It facilitated effective loading of AMO-21 and prevented its degradation under 50% FBS (Figure 2C and Table 2), suggesting similar mechanism in the vascular and tumor microenvironment. In addition to stability under aqueous environments, a sustained release of AMO-21 *in vitro* suggests diffusion from the polymeric matrix of NPs for effective gene silencing (Figure 2D).

Nonspecific interaction between nanocarriers and non-target cells is a critical challenge that limits the therapeutic efficacy resulting in adverse effects [30]. Using fluorescently-labeled AMO-21 and FAM-AMO-21 (green) encapsulated rhodamine B (red)-labeled HER-PEG-PCL NPs, we monitored the intracellular uptake of AMOs using fluorescence microscopy. FAM-AMO-21 was visualized in the cytoplasm of GC cells after transfection. Compared with PEG-PCL, the HER2 targeting ligand in the formulation contributed to improved cellular uptake, and enhanced transfection efficiency of NUGC4 cells (Figure 3B), via recognition of overexpressed surface antigens. Furthermore, we established the targeted uptake using HER2-negative cell line SGC7901 as a control. We found that the cellular uptake of HER-PEG-PCL and PEG-PCL NPs was similar (Figure 3A). The reduced uptake level of FAM-AMO-21-TNPs in SGC7901 cells than in NUGC4 cells further confirmed that the entry of TNPs into GC cells was mediated by trastuzumab antibody-mediated endocytosis (Figure 3A, 3B). The cellular effect of the nanoformulation containing trastuzumab conjugated with nanocarriers was enhanced by active entry into cancer cells expressing HER2.

Trastuzumab is cytotoxic even after conjugation with NPs, as shown by SDS-PAGE (Supplementary Figure 3). Trastuzumab conjugated with PEG-PCL NPs showed cytotoxic effects similar to free trastuzumab against NUGC4, which suggests that the antibody activity was retained even after nanoparticle synthesis. The AMO-21-TNPs showed a higher level of cytotoxicity and apoptosis compared with trastuzumab, trastuzumab + AMO-21-NPs or trastuzumab + AMO-21-Lipofectamine 2000 (Figure 4A, 4B, and 4C). These results indicate that AMO-21-TNPs penetrate the cells and release AMO-21. The findings suggest that the enhanced cytotoxicity of AMO-21-TNP in NUGC4 cells was mediated via targeted delivery. The cellular interaction and increased transfection efficiency significantly down-regulate miR-21 expression compared with PEG-PCL NPs and Lipofectamine 2000 in NUGC4 cells, as demonstrated by real-time PCR. The expression of PTEN is increased continuously and the AMO-21-TNPs suppression of NUGC4 cell proliferation and growth is enhanced (Figure 4A, 4B, and 4C) consistent with previous studies in GC cells with high miR-21 expression restoring sensitivity to trastuzumab [13]. Therefore, trastuzumab acts as a targeting ligand to improve the transfection rate of AMO-21 in NUGC4 cells. Furthermore, TNPs treatment showed no cytotoxicity toward SGC7901 cells (Figure 4D, 4E and 4F), which showed a potential for reduced systemic toxicity while retaining efficacy against target cells. The efficacy of trastuzumab-conjugated NPs was higher and more direct compared with PEG-PCL NPs alone for HER2-positive cells, with less cytotoxicity for HER2-negitive cells. Therefore, increased anti-tumor effect in HER2-positive GC cells decreases the incidence of side effects. Notably, none of the groups showed any cytotoxicity toward SGC7901 except trastuzumab + AMO-21-Lipofectamine 2000. Although the AMO-21-Lipofectamine 2000 induced a response against miR-21 similar to AMO-21-NPs in NUGC4, the inhibitory effect of trastuzumab + AMO-21-Lipofectamine 2000 was statistically higher than that of trastuzumab combined with AMO-21-NPs. The cytotoxicity of Lipofectamine 2000 for GC cells (Figure 4D, 4E and 4F) can be attributed to its potent cationic charge. Therefore, positively-charged nanoparticles reported in this study are not cytotoxic to HER2-negitive cells, and therefore, represent potential clinical value.

Compared with a commercial transfection agent lipofectamine 2000 and PEG-PCL NPs, the TNP encapsulating AMO-21 led to the efficient inhibition of miR-21 and an increase in PTEN expression in NUGC4 cells, while the treatment of free AMO-21, resulted in negligible change (Figure 6A, 6B). This suggests that the inhibition of miR-21 and subsequent increment in target genes can be attributed to the antagonism of miR-21 with AMO-21. Our results are consistent with previous reports suggesting that the microRNA-21/PTEN pathway regulates the sensitivity of HER2-Positive gastric cancer cells to trastuzumab [13]

Reports investigating targeted PEG-PCL-based delivery combined with ligands for anti-miR delivery to GC are rare. In this study, we reported the synthesis of PEG-PCL NPs targeting HER2 for the delivery of AMO-21 to GC cells. The TNPs showed favorable physicochemical properties and selective uptake by targeted cells. TNPs containing AMO-21 significantly enhanced target genes that are known to be repressed by miR-21. These findings suggest the potential application of anti-miR therapies in GC. These studies represent the initial step in AMO-21 and nanotechnology-based therapies against gastric cancer. In addition, the potential role of HER-PEG-PCL NPs to transfect AMOs, as well as therapeutic antibodies into cancer cells facilitates a multi-pronged strategy against trastuzumab-resistant tumors. The efficacy of AMO-21 TNPs in targeting gastric tumors will be investigated in future studies.

## MATERIALS AND METHODS

### Synthesis of PEG-PCL and HER-PEG-PCL copolymers

The PEG-PCL copolymers were prepared via ring opening copolymerization as described previously [23]. A single-step carbodiimide coupling with EDAc and Sulfo-NHS in aqueous phase was used to develop conjugates of trastuzumab [21]. Briefly, the stock solution (1 mg/mL) was prepared by diluting trastuzumab in borate buffer (pH 8.4). Dry PEG-PCL NPs was incubated with trastuzumab in borate buffer and supplemented with EDAc and Sulfo-NHS. The free primary amino groups on the NPs surface were conjugated with the carboxylic groups on the antibodies. After incubation overnight at room temperature under gentle end-to-end mixing, followed by centrifugation, the NPs were obtained. NPs were washed twice with borate buffer. The pellets were treated with ultrapure water for further analysis. The supernatant was scanned for antibody concentration at 595 nm using an ELISA plate Reader (Synergy HT, BioTek Instruments Inc., Winooski, Vermont). The antibody levels on the NPs surface were determined by subtracting the levels in the supernatant from the original concentration.

### Preparation of AMO-21-loaded nanoparticles

**Figure 7:**
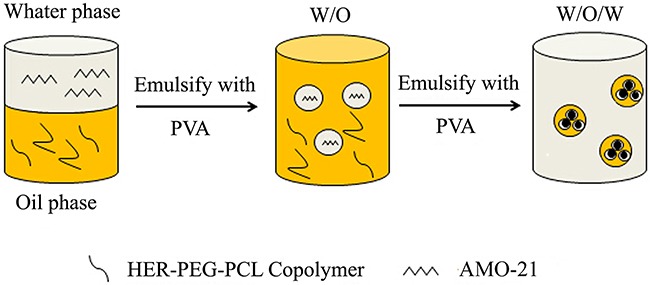
The scheme of AMO-21-HER-PEG-PCL NPs by a double-emulsion solvent evaporation technique (PVA: polyvinyl alcohol; AMO-21: anti-miR-21 oligonucleotide)

The AMO-21(GenePharm, Shanghai, China) containing PEG-PCL or HER-PEG-PCL nanoparticles were developed by a double-emulsion solvent evaporation technique according to published methods [29]. In brief, AMO-21 or FAM-AMO-21 combined with spermidine in a 10:1 molar ratio of polyamine nitrogen to polynucleotide phosphate (N/P ratio) was dissolved in RNAse- and DNAse-free phosphate buffered saline (PBS) (0.01 M, pH 7.4). Polyamine complexes containing AMO-21 or FAM-AMO-21 were developed at room temperature for 15 min on a rotary shaker. This aqueous solution was transformed into an emulsion by mixing with a copolymer (3 mg) dissolved in dichloromethane (100 mL). This mixture was developed into a double emulsion by combining with 5% polyvinyl alcohol (PVA) followed by sonication. The double emulsion was transferred into a beaker containing aqueous 0.3% (v/v) PVA (Figure 7). The cocktail was stirred for 3 h to allow evaporation of dichloromethane and harden the particles. NPs were obtained by centrifugation. They were washed, rapidly frozen, and lyophilized. The supernatant was used to measure the antibody concentrations by ELISA as described previously.

### Surface chemistry

The presence of trastuzumab on NPs surface was determined by X-ray photoelectron spectroscopy (XPS). The NPs surface was further analyzed in terms of the specific binding energy (eV) of the elements. The eV was recorded from 0 to 1000 eV, with pass energy of 80 eV under fixed transmission. Nitrogen was analyzed under fine mode with 0.5 eV. The data were processed using specific XPS software.

### Size, surface charge, encapsulation and drug loading measurement efficiency

The size, dispersity, zeta potential, and morphology of NPs were determined. Dynamic light scattering (DLS) (Brookhaven Instruments Corporation, USA) was employed to measure the hydrodynamic size and polydispersity. Zeta potential was measured using Zetaplus (Brookhaven Instruments Corporation, USA). The samples were stored at 37°C in PBS. The morphology of NPs was analyzed using transmission electron microscopy (TEM, JEM-100S, JEOL, Japan). A single drop of diluted NP suspension on a copper grid covered with nitrocellulose membrane was air-dried at room temperature and negatively stained with phosphotungstic sodium solution (1% w/v). Encapsulation efficiency (EE) and drug loading content (DLC) of AMO-21 were determined [29]. In brief, AMO-21 NPs (15 mg) were dissolved in dichloromethane (0.5 mL) at room temperature for 30 min, followed by extraction of AMO-21 from the organic phase using twice the volume of TE buffer (10 mM Tris e HCl, 1 mM EDTA, pH 7.4). After vortexing vigorously, the mixture was centrifuged at 12,000 rpm and 4°C for 10 min. The AMO-21 level was analyzed using the QuantiT^TM^, PicoGreen^TM^ assay according the manufacturer's instructions (Invitrogen, USA). The DLC and EE were calculated by the followingequations:

$$\text{DLC}\%=\frac{\text{Weight of the drug in nanoparticles}}{\text{Weight of the drug - loaded nanoparticles}}\times 100\%$$

$$\text{EE}\%=\frac{\text{Weight of the drug in nanoparticles}}{\text{Weight of the feeding drug}}\times 100\%$$

### SDS-PAGE

The HER2 antibody conjugated with NP was subjected to SDS-PAGE and compared with native HER2 antibody. SDS-PAGE was performed under reducing conditions at a constant voltage of 200 V in a Tris/glycine/SDS buffer. The gels were silver-stained to identify the protein. The gels were destained and dried.

### Structural stability, AMO-21 protection and release from TNPs *in vitro*

NPs, TNPs, AMO-21-NPs, AMO-21-TNPs were stored at 37°C immersed in phosphate buffered saline (PBS). The molecular size was monitored for 16 days for structural stability. The serum stability assay indicated protective ability of TNPs against nuclease degradation. Briefly, AMO-21-NPs, AMO-21-TNPs and free AMO-21 were treated with 50% fetal bovine serum (FBS) at 37°C for various intervals of time. Aliquots of each sample were loaded onto a 1.5% (w/v) agarose gel containing ethidium bromide. The release of AMO-21-TNPs and AMO-21-NPs was determined by dissolving a specific amount of NPs in RNAse- and DNAse-free PBS (0.01 M, pH 7.4) and transferred into a dialysis bag (MWCO 3500 Da). The dialysis bag was immerged in 5 mL release medium under gentle agitation at 37°C for 96 h. At predetermined time points, the release medium was replaced by an equivalent release medium. The AMO-21 concentration was analyzed using QuantiT^TM^, PicoGreen^TM^ assay.

### Cell culture

NUGC4, NCI-N87, SGC7901 and BGC823 were supplied by American Type Culture Collection (ATCC). We evaluated HER2 expression level in the cells by Western blot and immunohistochemistry as previously described [31]. HER2 was expressed positively in NCI-N87 and NUGC4 cells, and negatively in SGC7901 and BGC823, consistent with previous studies [13, 32] (Supplementary Figure 2). The miR-21 expression in the cells showing HER2-positive expression was determined by real-time reverse-transcription polymerase chain reaction (RT-PCR) [13]. The expression of miR-21 was higher in NUGC4 SGC7901 than in NCI-N87 BGC823 cells (Supplementary Figure 3), which were therefore, selected for further study. All cells were cultured in RPMI 1640 medium, supplemented with 10% fetal bovine serum and incubated at 37°C at 5% CO2 and 95% humidity.

### Cellular uptake by fluorescence microscopy

Rhodamine B-labeled particles were reacted with hydroxyl group in HER-PEG-PCL and PEG-PCL copolymers as previously described [33]. The labeled NPs were loaded with FAM-labeled AMO-21 (FAM-AMO-21) and the fluorescent signals were used to track the location of AMO-21 and NPs in NUGC4 and SGC7901 cells. Cells were loaded in 6-well plates at a density of 2×10^5^ cells/well and incubated overnight. An additional incubation for 2 h was performed using a fixed concentration of FAM-AMO-21-loaded Rhodamine B-labeled HER-PEG-PCL and PEG-PCL NPs. The cells were washed three times with PBS, and fixed with 4% paraformaldehyde (PFA) for 30 min at room temperature. Following another round of washing the media were replaced with fresh culture media. The fluorescence was determined using an Olympus LX71epifluorescence microscope (Olympus, Tokyo, Japan). For quantification, the color change in the PEG-PCL and HER-PEG-PCL groups was detected by the naked eye and analyzed using image analysis software (ImageJ). The integrated optical density (IOD) was used to quantify the fluorescence. The IOD of Rhodamine B and FAM were evaluated by performing tests under identical experimental conditions, in triplicate

### Cell proliferation

The cytotoxic effects of NPs on NUGC4 and SGC7901 were studied using MTT assay as previously described. The cells were layered at a density of 4000 cells/well in 96-well plates. After 24 h, the culture medium was replaced with specific concentrations of saline, trastuzumab, blank TNPs, AMO-21-Lipofectamine 2000, + trastuzumab AMO-21-NPs + trastuzumab and AMO-21-TNPs. Cell proliferation was determined at 490 nm.

### Apoptosis

NUGC4 and SGC7901 cells were incubated overnight in 6-well culture plates at a density of 1 × 10^5^ cells/well, and treated differently to analyze cell proliferation. The different groups were as follows: saline control, blank NPs, blank TNPs, free AMO-21, trastuzumab, AMO-21-Lipofectamine 2000 + trastuzumab, AMO-21-NPs + trastuzumab, AMO-21-TNPs for72 h. The cells were processed as described in the cell apoptosis kit (Alexa Fluor 488 annexin V/Dead Cell Apoptosis Kit with Alexa Fluor 488 annexin V and PI for Flow Cytometry, Invitrogen), followed by flow cytometry (BD Biosciences, Sparks, MD, USA).

### The real-time biodistribution of TNPs in gastric cancer xenograft mice

The real-time biodistribution of the NPs was investigated by NIRF imaging. The copolymer was tagged with NIR-797-isothiocyanate to track the position of the particles. Briefly, the NIR-797-isothiocyanate and copolymers were dissolved in DMF and stirred at room temperature for 8 h. Any unconjugated NIR-797-isothiocyanate was removed by dialysis (MWCO 3500 Da) for 2 days. The solution of NIR-797-labeled TNPs was lyophilized until further use. NIR-797-labeled TNPs (equivalent to the 10 mg/kg dose of trastuzumab used in the antitumor study) were intravenously administrated into NUGC4 tumor-bearing mice. The real-time biodistribution of the NPs in the tumor-bearing mice was visualized using an IVIS Lumina system (Xenogen Co., Alameda, CA, USA) at 1 h, 4 h, 8 h, 24 h, 48 h, 72 h, 96 h, 120 h, and 144 h post-intravenous administration. NIRF data were collected at 745 nm, with the exposure time set to 2 s. At 144 h after injection, the tumors and main organs, including the heart, lungs, spleen, liver, kidneys, intestines, stomach, and brain, were excised for *ex vivo* imaging.

### *In vivo* antitumor effect of AMO-TNPs

5 × 10^6^ NUGC4 cells in 0.1 ml RPMI 1640 medium were implanted subcutaneously into the right posterior flanks of BALB/c nude mice (male, 4–5 weeks old). The tumor volume was calculated using the formula W × L^2^/2, where W is the widest diameter, and L is the longest diameter. When 80% of the tumors reached a volume of 100 mm^3^, mice were randomly grouped into treatment cohorts, each of which contained 5 mice. Tumor volumes were calculated using the formula: (mm^3^) = (L × W^2^) × 0.5. The tumor volume variation was measured in tumor-bearing mice that were treated intravenously withsaline control (2 times/week × 3 weeks), blank NPs (2 times/week × 3 weeks), trastuzumab (10 mg/kg, 2 times/week × 3 weeks), TNPs (10 mg/kg trastuzumab eq., 2 times/week × 3 weeks), AMO-21-NPs (2 times/week × 3 weeks), and AMO-21-TNPs (10 mg/kg trastuzumab eq., 2 times/week × 3 weeks). The tumors were measured every 3 days until 21 days after treatment. Relative tumor volumes were calculated using the following equation, to reduce the impact of differences in initial tumor volume after grouping:

$$\text{relative tumor volume}=\frac{V}{V0}\times 100\%$$

V is the absolute tumor volume, and V0 was the average tumor volume of the group on Day 1

### Real-time reverse-transcription polymerase chain reaction (RT-PCR)

Saline controls of NUGC4 cells were prepared. The cells were also treated with AMO formulations (free AMO-21, AMO-21-NPs, AMO-21-Lipofectamine 2000, AMO-21-TNPs) and incubated for an additional 2 days. The total RNA extracted from the cells using an RNA isolation kit (Tian Gen) was stored at −70°C until use. The mRNA transcripts were measured with reference to RUN6B, using a SYBR Green qRT-PCR miRNA Detection Kit (TianGen, Bejing, China) and SYBR Green I according to the manufacturer's protocols [34]. The expression levels of PTEN were also quantified by SYBR Green qRT-PCR and normalized to beta-actin (ACTB) as published elsewhere. All of the primers are listed in Supplementary Table 1. The results were analyzed using the 2^(−ΔΔCT)^ comparative method [35]. All experiments were carried out in triplicate.

### Statistical analysis

All the experiments were performed in triplicate. Continuous variables were expressed as the mean ± SD. A Student's t-test was used to compare the differences between groups. A *P* value that was less than 0.05 indicated statistical significance.

## SUPPLEMENTARY MATERIALS FIGURES AND TABLES



## References

[1] Chen W, Zheng R, Baade PD, Zhang S, Zeng H, Bray F, Jemal A, Yu XQ, He J (2016). Cancer statistics in China, 2015. CA Cancer J Clin.

[2] Power DG, Kelsen DP, Shah MA (2010). Advanced gastric cancer--slow but steady progress. Cancer Treat Rev.

[3] Shah MA, Janjigian YY, Stoller R, Shibata S, Kemeny M, Krishnamurthi S, Su YB, Ocean A, Capanu M, Mehrotra B, Ritch P, Henderson C, Kelsen DP (2015). Randomized multicenter phase II study of modified docetaxel, cisplatin, and fluorouracil (DCF) versus DCF plus growth factor support in patients with metastatic gastric adenocarcinoma: a study of the US gastric cancer consortium. J Clin Oncol.

[4] Gravalos C, Jimeno A (2008). HER2 in gastric cancer: a new prognostic factor and a novel therapeutic target. Ann Oncol.

[5] Hofmann M, Stoss O, Shi D, Buttner R, van de Vijver M, Kim W, Ochiai A, Ruschoff J, Henkel T (2008). Assessment of a HER2 scoring system for gastric cancer: results from a validation study. Histopathology.

[6] Tanner M, Hollmen M, Junttila TT, Kapanen AI, Tommola S, Soini Y, Helin H, Salo J, Joensuu H, Sihvo E, Elenius K, Isola J (2005). Amplification of HER-2 in gastric carcinoma: association with Topoisomerase IIalpha gene amplification, intestinal type, poor prognosis and sensitivity to trastuzumab. Ann Oncol.

[7] Bang YJ, Van Cutsem E, Feyereislova A, Chung HC, Shen L, Sawaki A, Lordick F, Ohtsu A, Omuro Y, Satoh T, Aprile G, Kulikov E, Hill J (2010). Trastuzumab in combination with chemotherapy versus chemotherapy alone for treatment of HER2-positive advanced gastric or gastro-oesophageal junction cancer (ToGA): a phase 3, open-label, randomised controlled trial. Lancet.

[8] Calin GA, Croce CM (2006). MicroRNA signatures in human cancers. Nat Rev Cancer.

[9] Kasinski AL, Slack FJ (2011). Epigenetics and genetics. MicroRNAs en route to the clinic: progress in validating and targeting microRNAs for cancer therapy. Nat Rev Cancer.

[10] Li Z, Rana TM (2014). Therapeutic targeting of microRNAs: current status and future challenges. Nat Rev Drug Discov.

[11] Deng Y, Huang Z, Xu Y, Jin J, Zhuo W, Zhang C, Zhang X, Shen M, Yan X, Wang L, Wang X, Kang Y, Si J, Zhou T (2014). MiR-215 modulates gastric cancer cell proliferation by targeting RB1. Cancer Lett.

[12] Ma L, Reinhardt F, Pan E, Soutschek J, Bhat B, Marcusson EG, Teruya-Feldstein J, Bell GW, Weinberg RA (2010). Therapeutic silencing of miR-10b inhibits metastasis in a mouse mammary tumor model. Nat Biotechnol.

[13] Eto K, Iwatsuki M, Watanabe M, Ida S, Ishimoto T, Iwagami S, Baba Y, Sakamoto Y, Miyamoto Y, Yoshida N, Baba H (2014). The microRNA-21/PTEN pathway regulates the sensitivity of HER2-positive gastric cancer cells to trastuzumab. Ann Surg Oncol.

[14] Weiler J, Hunziker J, Hall J (2006). Anti-miRNA oligonucleotides (AMOs): ammunition to target miRNAs implicated in human disease?. Gene Ther.

[15] Lennox KA, Owczarzy R, Thomas DM, Walder JA, Behlke MA (2013). Improved performance of anti-miRNA oligonucleotides using a novel non-nucleotide modifier. Mol Ther Nucleic Acids.

[16] Ge X, Zhang Q, Cai Y, Duan S, Chen S, Lv N, Jin T, Chen Y, Yuan W (2014). PEG-PCL-DEX polymersome-protamine vector as an efficient gene delivery system via PEG-guided self-assembly. Nanomedicine (Lond).

[17] Shukla GC, Haque F, Tor Y, Wilhelmsson LM, Toulme JJ, Isambert H, Guo P, Rossi JJ, Tenenbaum SA, Shapiro BA (2011). A boost for the emerging field of RNA nanotechnology. ACS Nano.

[18] Zhang M, Zhou X, Wang B, Yung BC, Lee LJ, Ghoshal K, Lee RJ (2013). Lactosylated gramicidin-based lipid nanoparticles (Lac-GLN) for targeted delivery of anti-miR-155 to hepatocellular carcinoma. J Control Release.

[19] Hatakeyama H, Murata M, Sato Y, Takahashi M, Minakawa N, Matsuda A, Harashima H (2014). The systemic administration of an anti-miRNA oligonucleotide encapsulated pH-sensitive liposome results in reduced level of hepatic microRNA-122 in mice. J Control Release.

[20] Lee H, Lytton-Jean AK, Chen Y, Love KT, Park AI, Karagiannis ED, Sehgal A, Querbes W, Zurenko CS, Jayaraman M, Peng CG, Charisse K, Borodovsky A (2012). Molecularly self-assembled nucleic acid nanoparticles for targeted in vivo siRNA delivery. Nat Nanotechnol.

[21] Liu Y, Li K, Liu B, Feng SS (2010). A strategy for precision engineering of nanoparticles of biodegradable copolymers for quantitative control of targeted drug delivery. Biomaterials.

[22] Haun JB, Devaraj NK, Hilderbrand SA, Lee H, Weissleder R (2010). Bioorthogonal chemistry amplifies nanoparticle binding and enhances the sensitivity of cell detection. Nat Nanotechnol.

[23] Li R, Li X, Xie L, Ding D, Hu Y, Qian X, Yu L, Ding Y, Jiang X, Liu B (2009). Preparation and evaluation of PEG–PCL nanoparticles for local tetradrine delivery. Int J Pharm.

[24] Iyer AK, Khaled G, Fang J, Maeda H (2006). Exploiting the enhanced permeability and retention effect for tumor targeting. Drug Discov Today.

[25] Croce CM (2009). Causes and consequences of microRNA dysregulation in cancer. Nat Rev Genet.

[26] Das M, Mohanty C, Sahoo SK (2009). Ligand-based targeted therapy for cancer tissue. Expert Opin Drug Deliv.

[27] Maeda H, Wu J, Sawa T, Matsumura Y, Hori K (2000). Tumor vascular permeability and the EPR effect in macromolecular therapeutics: a review. J Control Release.

[28] Cirstoiu-Hapca A, Buchegger F, Lange N, Bossy L, Gurny R, Delie F (2010). Benefit of anti-HER2-coated paclitaxel-loaded immuno-nanoparticles in the treatment of disseminated ovarian cancer: therapeutic efficacy and biodistribution in mice. J Control Release.

[29] Liu Q, Li RT, Qian HQ, Wei J, Xie L, Shen J, Yang M, Qian XP, Yu LX, Jiang XQ, Liu BR (2013). Targeted delivery of miR-200c/DOC to inhibit cancer stem cells and cancer cells by the gelatinases-stimuli nanoparticles. Biomaterials.

[30] Whitehead KA, Langer R, Anderson DG (2009). Knocking down barriers: advances in siRNA delivery. Nat Rev Drug Discov.

[31] Zhang Z, Wang J, Ji D, Wang C, Liu R, Wu Z, Liu L, Zhu D, Chang J, Geng R, Xiong L, Fang Q, Li J (2014). Functional genetic approach identifies MET, HER3, IGF1R, INSR pathways as determinants of lapatinib unresponsiveness in HER2-positive gastric cancer. Clin Cancer Res.

[32] Cui Y, Li SB, Peng XC, Wu J, Fu GH (2015). Trastuzumab inhibits growth of HER2-negative gastric cancer through gastrin-initialized CCKBR signaling. Dig Dis Sci.

[33] Liu Q, Li RT, Qian HQ, Yang M, Zhu ZS, Wu W, Qian XP, Yu LX, Jiang XQ, Liu BR (2012). Gelatinase-stimuli strategy enhances the tumor delivery and therapeutic efficacy of docetaxel-loaded poly(ethylene glycol)-poly(varepsilon-caprolactone) nanoparticles. Int J Nanomedicine.

[34] Shi SJ, Zhong ZR, Liu J, Zhang ZR, Sun X, Gong T (2012). Solid lipid nanoparticles loaded with anti-microRNA oligonucleotides (AMOs) for suppression of microRNA-21 functions in human lung cancer cells. Pharm Res.

[35] Livak KJ, Schmittgen TD (2001). Analysis of relative gene expression data using real-time quantitative PCR and the 2(−Delta Delta C(T)) method. Methods.

