# Supplementary material for: Enhanced antiproliferative activity of antibody-functionalized polymeric nanoparticles for targeted delivery of anti-miR-21 to HER2 positive gastric cancer
Source: Oncotarget. 2017 May 22;8(40):67189–202. doi: 10.18632/oncotarget.18066 (PMC5620166; doi:10.18632/oncotarget.18066)
Supplement: Supplementary file 1 [file oncotarget-08-67189-s001.pdf]

Enhanced antiproliferative activity of antibody-functionalized polymeric nanoparticles for targeted delivery of anti-miR-21 to HER2 positive gastric cancer

SUPPLEMENTARY MATERIALS

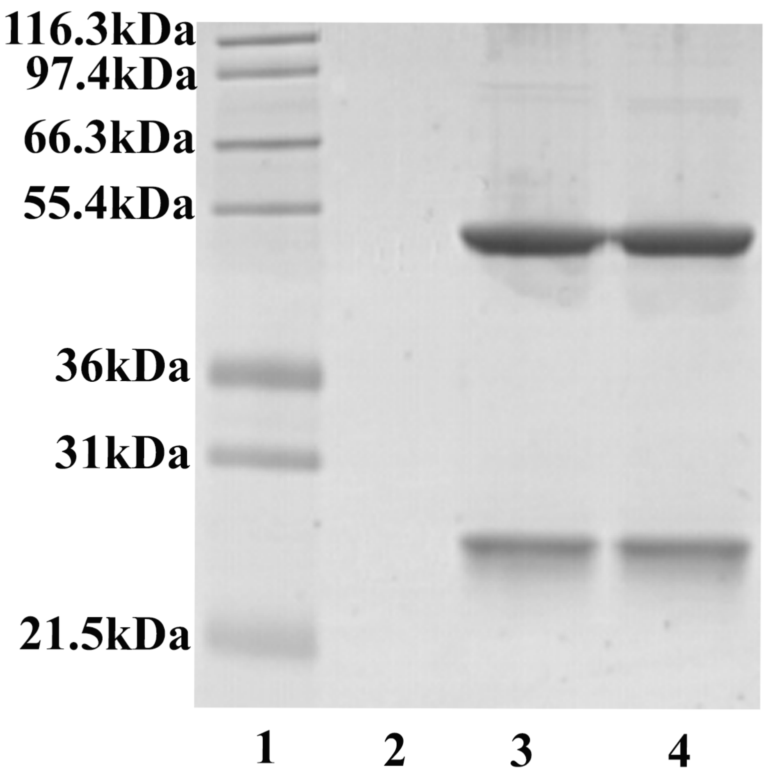

Supplementary Figure 1: SDS-PAGE of HER2 antibody: Lane 1 represents the molecular weight markers; Lane 2 represents the blank control; Lane 3 represents the native trastuzumab control; and Lane 4 represents the HER-PEG-PCL NPs coated with trastuzumab. (SDS-PAGE: sodium dodecyl sulfate polyacrylamide gel electrophoresis; HER2: human epidermal growth factor receptor 2).

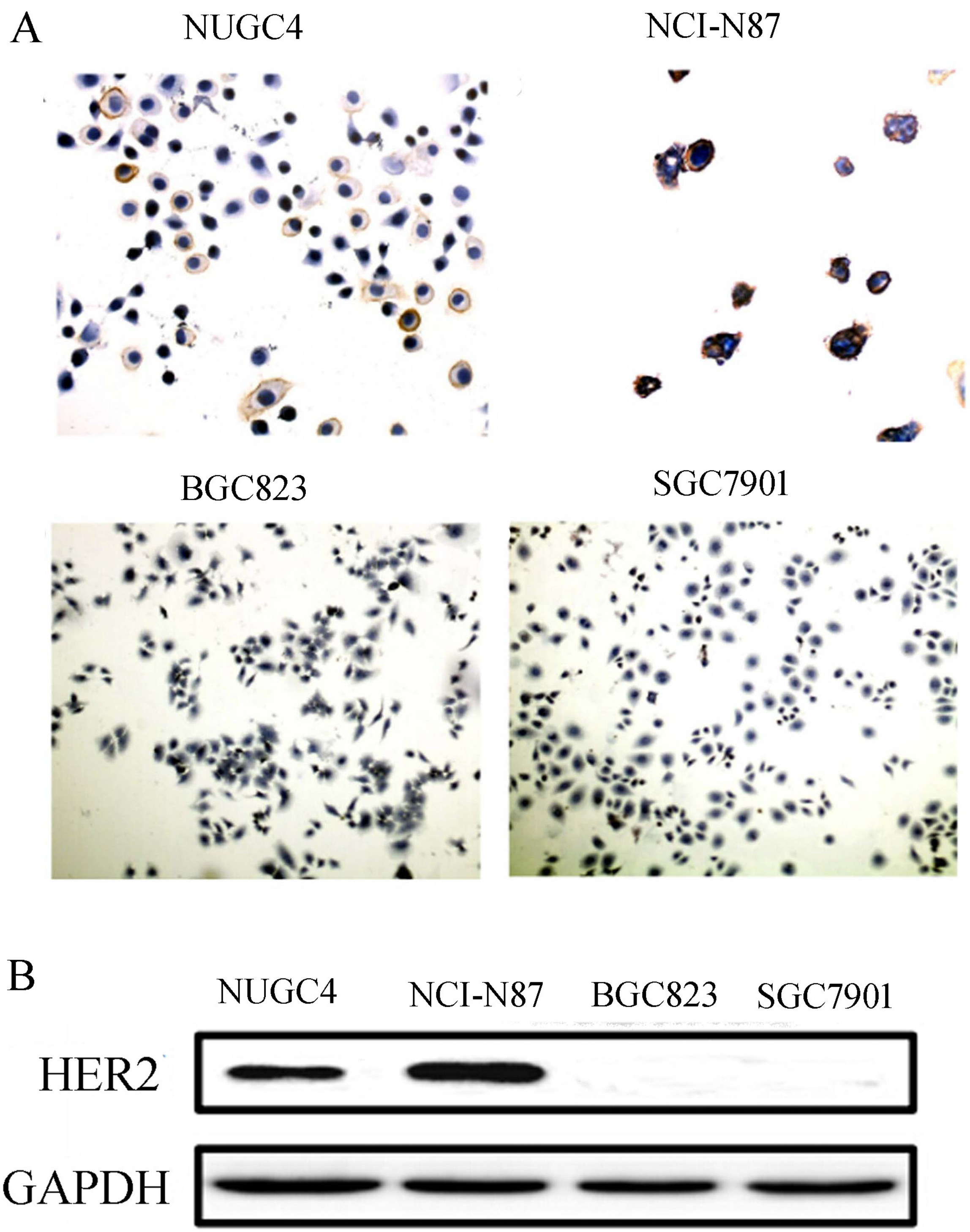

Supplementary Figure 2: Detection of HER2 expression. Immunohistochemical **(A)** and westen-blot **(B)** analysis of HER2 expression in NUGC4, NCI-N87, BGC823 and SGC7901 cells. (HER2, human epidermal growth factor receptor 2).

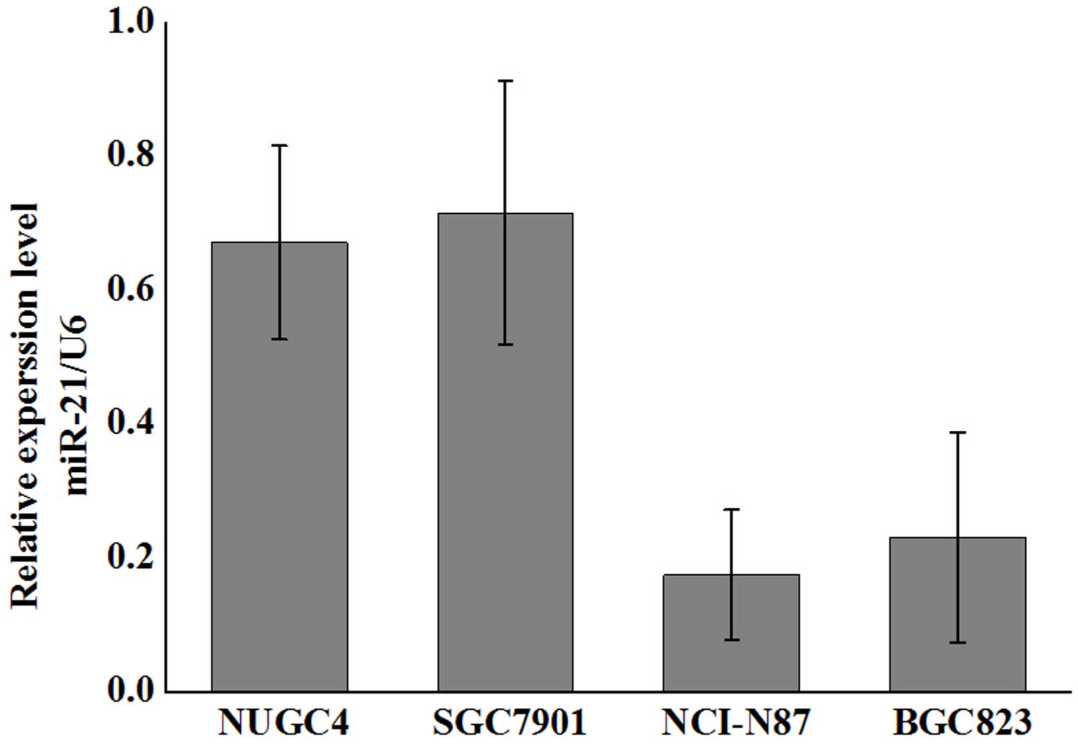

Supplementary Figure 3: Expression of miR-21 in NUGC4, NCI-N87, BGC823 and SGC7901 cells was detected by RT-qPCR. (RT-qPCR, real-time reverse-transcription polymerase chain reaction).

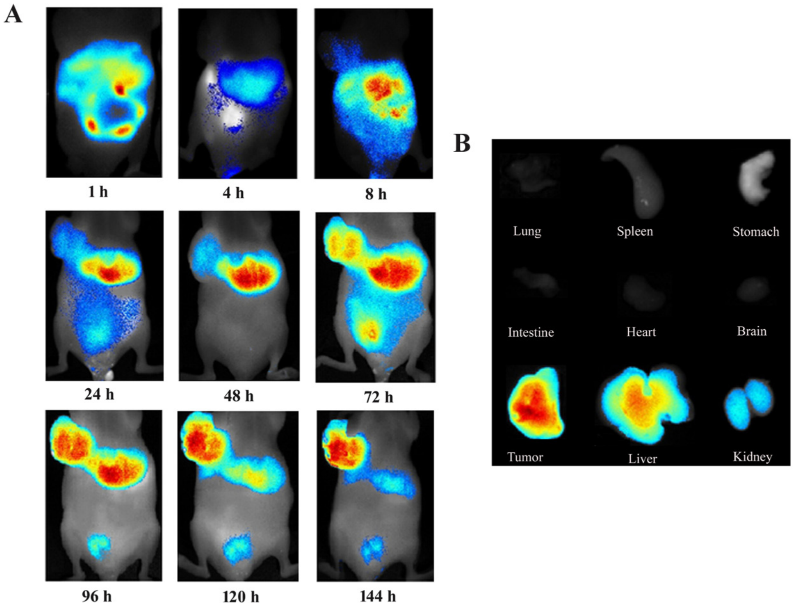

Supplementary Figure 4: The real-time biodistribution of NIR-797 labeled TNPs in NUGC4 gastric tumor-bearing mice. (A) NIR fluorescence signals were observed in both the tumors and the abdomen. **(B)** NIR fluorescence signals were observed in tumor, heart, brain, liver, spleen, kidneys, stomach, intestines, and the lungs respectively.

Supplementary Table 1: Oligonucleotide and primer sed for qRT-PCR analysis

**Oligonucleotide sequences for miRNA modulation**	
AMO-21	TCATGATCA GACTGATAAGCTA
Primers used for qPCR	
miR-21_forward	ACACTCCAGCTGGGTAGCTTATCAGACTGA
miR-21_reverse	TGGTGTCGTGGAGTCG
PTEN_forward	CCTTCTCCATCTCCTGTGTAATCAA
PTEN_reverse	GTTGACTGATGTAGGTACTAACAGCAT
U6 _forward	CTCGCTTCGGCAGCACA
U6 _reverse	AACGCTTCACGAATTTGCGT
GAPDH_forward	AGCCACATCGCTCAGACAC
GAPDH_reverse	GCCCAATACGACCAAATCC

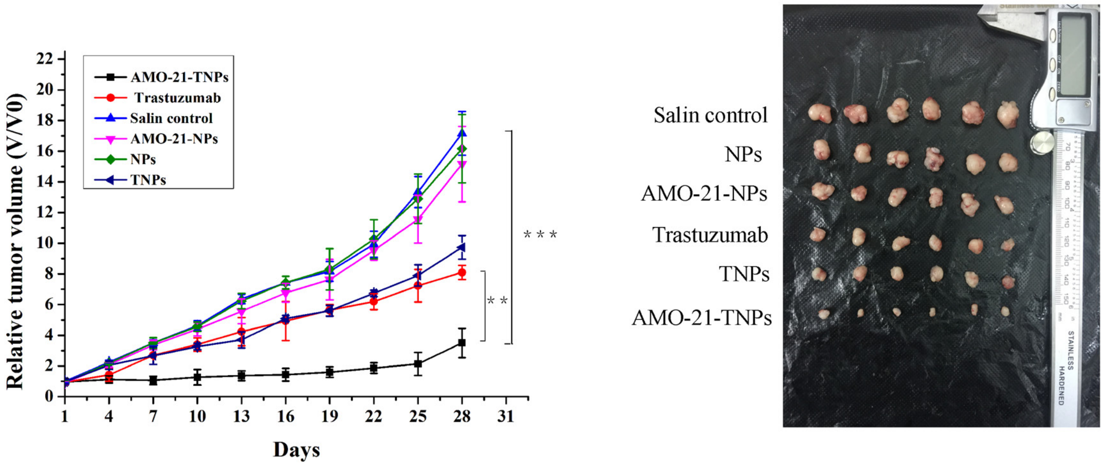

Supplementary Figure 5: Inhibition of NUGC4 xenograft tumor growth by AMO-21TNPs in comparison with various formulations (n = 6). **, $P < 0.01$ compared with trastuzumab group.***, $P < 0.001$ compared with control group.

Supplementary Table 2: Integrated optical density (IOD) of Rhodanmin-B-PEG-PCL, Rhodanmin-B-HER-PEG-PCL NPs, FAM-AMO-21-PEG-PCL NPs and, FAM-AMO-21-HER-PEG-PCL in SGC7901 and NUGC4 cells using ImageJ software

Variable (IOD) [a]	PEG-PCL NPs [b]	HER-PEG-PCL NPs	p
Rhodanmin-B (SGC7901)	57901 ± 573.3	58962 ±1512.24	>0.05
FAM (SGC7901)	41629 ± 1527.5	43465 ± 1000	>0.05
Rhodanmin-B (NUGC4)	58939 ± 739.2	90281 ± 996.7	<0.001*
FAM (NUGC4)	43143 ± 871.9	122773 ± 2300	<0.001*

[a] IOD: integrated optical density.
[b] The SD value was for the mean IOD obtained from the three measurements.

Supplementary Table 3: Integrated optical density (IOD) of Rhodanmin-B -PEG-PCL NPs and FAM-AMO-21-PEG-PCL NPs in SGC7901 and NUGC4 cells using ImageJ software

Variable (IOD)[a]	SGC7901[b]	NUGC4[b]	p
Rhodanmin-B (PEG-PCL NPs)	57901 ± 573.3	58939 ± 739.2	>0.05
FAM (HER-PCL NPs)	43143 ± 871.9	41629 ± 1527.5	>0.05

[a] IOD: integrated optical density.
[b] The SD value was for the mean IOD obtained from the three measurements.
